# Abstracts and Gleanings

**Published:** 1885-06-20

**Authors:** 


### ABSTRACTS AND GLEANINGS.

**Obstetric Forceps.**—A writer in New York Medical Times thus writes in regard to using the Obstetric Forceps:

It matters very little what forceps we choose—every practitioner has his preference. My own is for the Elliot long, and the Sawyer short, although the forceps of Dr. Comstock, of St. Louis, are most excellent. Many new inventions in forceps are constantly appearing. Tarnier and Lusk's modification of them are very useful in particular cases, but they can hardly be advised for the young obstetrician who desires one, or at the most, two instruments.

Your forceps have been selected—they are ready to your hand—you are confronted with a case that calls for their use—when should they be applied, and how?

The general rules for instrumental interference are variously laid down in works on obstetrics; but for our purpose we may reduce them to three:

*First*, When, from the size of the fœtal head and the contracted state of the pelvis, Nature alone could not accomplish delivery.

*Second*, When, from the enfeebled state or condition of the mother, from *any cause*, prompt and rapid delivery is necessary.

*Third*, When we can safely shorten the duration of labor, and mitigate its pains.

These are the three general rules I would advocate. The application of them must be learned by experience, and at the bedside of the patient.

The forceps are almost invariably applied to the head, and most frequently after its passage through the os uteri, constituting what is called the low operation, and in this condition of affairs, when we find that the pains are becoming less frequent, the patient exhausted, and the labor making no progress, we should no longer hesitate, but at once proceed to apply the forceps, and in the following manner: Place the woman in a comfortable position for herself, and as convenient for the operator as possible. Generally she should be crosswise of the bed, the knees drawn up and flexed, the heels resting on the sideboard, her head and shoulders supported by the nurse. First, warm the instruments by plunging them in hot water; wipe them dry, and anoint the blades with vaseline; then insert two fingers of the right hand into the vagina as a guide; with the left, grasp the lower blade and pass it gently, and the only direction I would give is, hold it easily and use no force, being careful only to have the concave surface on the left side of the head. It cannot go wrong: there is only one place for it to go, and if no force is used, and it is inserted during the absence of a pain, it will certainly find its way where it belongs. The canal is bathed in mucus, the head is smooth and globular, the blade warm and oily, and it would be an impossibility, unless brute force is used, to insert it any way but the right way. When the first blade is properly applied, drop the handle; no assistant is needed; it will stay where you have placed it, unless a pain occurs, when we should gently support the handle until it passes. Now, reversing the hands, insert the upper blade in the same manner, only being careful that it is placed on the right side of the head. Both blades being introduced, take the handles in your hands, and, by gentle manipulation, they will lock; if they do not, withdraw them slightly and try again, using no force; take *time*, and stop on the occurrence of a pain.

Your forceps are now locked. It will be proper to bring the handles together, to ascertain how much pressure to use. The screw in the handle of the Elliot and other forceps of like pattern is useful to regulate the pressure, and the bringing together of the handles will also inform you if any portion of the maternal tissues is caught by the blades.

Your forceps are now introduced. It is simple enough, and it is strange that so much has been written about so slight an operation, for any physician in ordinary cases, like the one I have described, with ordinary delicacy of touch, ought to find no difficulty in this procedure.

Now, with your left hand grasping the lower portion of the instruments just at the junction of the blades, the palm upwards, the index on the fœtal head, the right hand firmly grasping the handles palm downwards, you are ready for action. Wait for a pain and then draw steadily in the direction of the curve of the pelvis, not too strongly at first, and ceasing when the pain passes. You want to assist, not overcome nature. The older accoucheurs advised that the force exerted should be two-thirds lateral and one-third tractive; but we believe this to be bad practice, as it may bruise the soft parts of the mother, and does no good. As with each successive pain the head advances little by little and finally presses on the perineum, care must be taken to avoid laceration; the left index finger being the guide for the advancing head. As it emerges from the vulva the forceps should be gradually elevated until the handles lie almost on the abdomen of the mother; then carefully unlock and withdraw the instruments, and let nature complete the delivery. This is the method of using instruments in the so-called low operation, and when the head is not too large, the Sawyer forceps are the best I have used. They are light, easy of introduction, and, by the curve of blade and handle, act almost automatically.

To recapitulate: Use deliberation, no undue force, and observe great care when the head presses on the perineum.

**Vinegar in Post-Partum Hemorrhage.**—About ten years since, I attended a patient who had a most violent post-partum hemorrhage, so severe, indeed, that I began to despair of arresting it. I had not ergot with me, and ice was not procurable. I directed the attendant to give a wineglassful of pure brandy. The uterus, which was before flaccid, contracted instantaneously under my hand, and the bleeding ceased. On proceeding to give some more brandy, I discovered that the patient had been given vinegar instead of brandy. The effect was so marked that I inquired of the old midwife who was with me whether she had ever heard of vinegar being used before. She informed me that, in her part of the country, it was considered an excellent remedy, but that she had rarely, if ever, used it. When lecturing to a class of pupil-midwives shortly afterwards, I mentioned the case and recommended them most strongly to give the vinegar a trial in case of need. It seems to have escaped my memory until, about two years ago, the midwife at Queen Charlotte's Lying-in Hospital reminded me of my recommendation, and told me she had given vinegar repeated trials, and preferred it to ergot on account of its certain and instantaneous action. She was such a reliable and clever midwife, that I felt her testimony might be taken. Since then I have carefully questioned all my pupil-midwives as to its action, for until recently it was never used in the hospital. They all agree that, in their cases of hemorrhage in the out-patient department, where they were allowed to use vinegar, hemorrhage was arrested more quickly than in the hospital with ergot. It was not until recently that I had a good test-case. The patient belonged to a family of "flooders." Her mother and two of her near relations had bled to death. As soon as the child was born, she began to flood. I expelled the placenta, and gave a wineglassful of vinegar. The uterus, which was very flaccid and constantly dilating, at once contracted firmly under my hand; it did not again relax, although the hemorrhage continued to a moderate extent. At the end of fifteen minutes, I gave a second dose, about two-thirds of a wineglassful. In both instances it was given pure, without any water. This soon arrested the hemorrhage, and the patient did well. I used no other means beyond holding the uterus, as I was perfectly satisfied with the result. I feel certain that I should not have obtained such favorable results with ergot. The action of vinegar is so rapid that I refrain from using it or permitting its use before the placenta is expelled, for fear of causing a retention of that body and making its removal difficult. From my own experience, and from the reports obtained from my midwives, pupil-midwives, and house-surgeons, I can confidently recommend the use of vinegar in post-partum hemorrhage. It is a remedy, if not always at hand, at any moment procurable, simple and harmless, not open to the objection against ergot, which, in the hands of midwives, is very liable to be used to hasten delivery, nor to the serious disadvantage and dangers of intra-uterine injections. If further trials, on a more extended scale, confirm my experience, I have no hesitation in saying that vinegar will have to be regarded as almost the specific for post-partum hemorrhage.—*W. C. Grigg, M.D., in British Medical Journal.*

**A Hair Tonic.**—The best remedy to stimulate the growth of hair, and to keep the hair from coming out, is uvedalia. If the scalp is dry and harsh, the ointment of uvedalia may be rubbed up with an equal part of vaseline and scented. If the sebaceous secretion is free, the tincture may be employed, adding bay rum, one or two parts. In either case the scalp is thoroughly rubbed with the remedy once or twice a day.—*Ec. Med. Journal.*

**Lobelia Inflata.**—One point to which I particularly wish to call attention is the use of small doses of lobelia frequently repeated. I believe that this is one of the most important uses of this drug. The size of the dose should be regulated by the susceptibility of the patient. Some patients are as susceptible to drop doses as others are to ten-drop doses. Children are usually very susceptible to the influence of lobelia. Five drops of tinct. lobelia and one drop of tinct. aconite in half a cup of water, given in teaspoonful doses every half-hour to infants, will be found very useful in those feverish conditions, attended with restlessness so common to such children. One drachm of the tinct. of lobelia with half a drachm of tinct. of aconite root in a glsss (℥vi) of water, given in teaspoonful doses every twenty or thirty minutes, is a good febrifuge for adults. Many hesitate to use lobelia, believing it to be a dangerous drug. With proper precaution, I believe there is no potent drug in the Materia Medica which is less dangerous. In some cases the drug needs to be given freely in order to obtain the desired effect. Sidney Ringer, in his *Therapeutics*, declares that this drug must be given in large doses in order to obtain satisfactory results. He says: "Unless given in large doses—doses considered by many, without foundation, as poisonous—the remedy is inoperative. Many erroneously consider that lobelia is a highly poisonous and dangerous drug, which must be given with much caution and close watching." In the diseases (asthma and whooping-cough) in which Binger recommends the use of lobelia, it is often necessary to push its action freely in order to obtain satisfactory results; but in many other affections, very gratifying results are obtained from small doses frequently repeated.—*Am. Med. Jour.*

**Vaccination during the Incubation of Variola.**—The mean duration of the incubation of variola is from eleven to twelve days. It seems to be shorter in the hemorrhagic form, then lasting but six or eight days, except that in the latter case the variations of its duration are simply *en rapport* with the gravity of the case. The beginning of the incubation is not prevented, or its progress modified, by the presence of the preliminary fever. Vaccination subsequently performed may prevent or modify the eruption. It is doubtful if, at this period, contagion may be conveyed by the atmosphere, but from the beginning of the first stage, the disease may be transmitted by inoculation. This last event presents itself particularly in the event of epidemic variola. To avoid it, it is preferable to use only *animal vaccine*, and one should abstain from vaccinating from arm to arm. It is better to collect the vaccine and not use the product until fifteen days after. Not only does vaccination prevent the spreading of smallpox, but it operates at the same time in a way to modify the eruption and to reduce the disease just as well after infection by variola as during the incubation. The result is often variable. It is often almost useless, when the vaccination has been made only three or four days previous to the reception of the poison of variola, and it is, therefore, better that one should commence it a longer period before the outbreak of the fever. When this last has made its appearance, it is necessary to have recourse to inoculation; for, no matter by what process protection is attempted, whether by scarifying or by subcutaneous injection, the progress of the disease and the gravity of the attack of the patient will be in no wise modified.—*M. Venay, in Revue de Medecine, Nov., 1884.*

**A New So-called Specific Treatment for Typhoid Fever.**—Dr. J. W. Hawkins thus writes to the *Kansas City Medical Record*, February, 1885: It is said by medical writers of the present day that there is no known specific treatment for typhoid fever. We are gravely told that "the abortive plan by the use of calomel is the only treatment that can be considered ætiological or casual." To this statement I respectfully demur. If calomel aborts the fever in fifteen to twenty days, the bromide of potassium treatment will do it in seven to ten days. The bromide of potassium is a medicine (unlike calomel) attended by no bad results, and upon it we can confidently rely. It may be given in any and all stages of the fever—first, second, third, fourth, fifth, or sixth week. If you see the patient on the first or last day of the fever, begin at once to administer the antidote—bromide of potassium. In the whole metasyncritic cycle of remedies for typhoid fever the bromide of potassium stands at the head. It accomplishes what no other known remedy has done, when properly administered. It usually arrests the fever in from seven to ten days after beginning its use. If the treatment is commenced at the beginning of the attack, five grain doses administered every three hours during the day only, and repeated daily, will usually be sufficient. But if in the last stage, from fifteen to forty grains will sometimes be required. In the last stage of a very severe case, when death seemed almost inevitable, I gave more than two hundred grains in twenty-four hours, producing no gastric disturbance whatever. The patient recovered. Hence, from this and other like cases, I am led to believe that we have a specific for enteric fever.

The truth of this has since been verified in the treatment of ten additional cases, the fever in every case being arrested in from seven to ten days. I think I am not talking too forcibly when I say that bromide of potassium is as much a specific for typhoid fever as the sulphate quinia is for (ague) intermittent fever.—*Med. and Surgical Reporter.*

**Recently** Dr. J. Bettelheim (*Wiener Med. Presse*, 1884, 45,) has tried the new local anæsthetic, muriate of cocaine, in a severe case of tenesmus of both rectum and bladder, and the result was very favorable and prompt, though not of more than a few hours' duration. Still, in the milder cases mentioned, where the tenesmus forms *the* symptoms for which the patient consults the physician, a transient action would be all that is necessary to insure success; and if the action of the cocaine in such cases can always be relied upon, on that one account alone the addition of the drug to our pharmacopœia would be a boon to many a sufferer. The solution employed in B.'s case was the usual 2 per cent. one.

**Monsel's Iron in Diarrhœa.**—Dr. Edward T. Williams says, in the *Boston Medical and Surgical Journal*, February 5, 1885:

"Ever since I began practice in 1868 I have been looking for a really satisfactory astringent in chronic catarrh of the bowels. There is, as every one knows, a class where the ordinary vegetable astringents fail to act, or at least act too feebly to do real good. The intestinal linings is in an ulcerous, or quasi-ulcerous, condition, and requires the potent action of a mineral astringent to treat it, as in cases of external ulcer. The acetate of lead is one of the best remedies in these cases, but cannot be safely given for any great length of time. Oxide of zinc, in pill form, is safe and efficient, but with children, who must take it in powder, often vomits and gripes. Sulphate of copper and nitrate of silver are still harsher, and for children quite out of the question. Subnitrate of bismuth is worse.

"I began trying, in 1876, at the Seashore Home, iron alum (the official sulphate of iron and ammonia). I found it better than anything I had previously tried, and have used it freely ever since. It is not quite so well borne by the stomach as lead and bismuth, but far better than zinc or copper. The dose for a child is from one to three grains; for adults, from three to ten. At the Seashore Home we make powders containing one grain of the salt to a twelfth of a grain of opium, giving one or more for a dose according to the age of the child. For adults the pill form is of course preferable. I have had the best results from its use.

"Last summer I began using Monsel's salt in its place, both in public and private practice. This I did from my experience of its great efficiency as a styptic, and a presumption that it might do equally well in diarrhœa, and have found it even better than iron alum. I have tried it only in the dry form, manufactured by Squib under the name of pulvis ferri, subsulphatis. In this State it is not officinal, though it is precisely the same as the officinal liquid ferri subsulphatis evaporated to dryness. It may be given in the same doses and in the same way as iron alum."—*Med. and Sur. Reporter*.

**Can Tuberculosis be Transmitted by Vaccination?**—In a recent number of the Centralb. fur Allgemeine Gesundheitspflege, Dr. Joseph Ackner gives an account of some experiments made by him with a view to determine whether, in a person suffering from tuberculosis and successfully vaccinated, the specific bacillus of tubercule is to be found in the lymph of the vaccine vesicle, so that there might be danger of transmitting tubercle if such lymph were used for vaccination. His conclusions are that in ordinary cases of consumption the vaccine lymph is entirely free from the tubercle bacillus, and that we have no reason to suppose that either scrofula or tubercle are propagated by vaccination.

It is not wise to use vaccine lymph taken from a scrofulous or unhealthy child, not so much because there is danger of transmitting scrofula, as because such lymph is unreliable and gives imperfect results which cannot be relied on as protection against small-pox.—*The Sanitary Engineer*, January 29.

**The officinal Aqua Creasoti**, or Creasote Water, is so important a preparation for one special use that it is well to notice it in order to emphasize that special use. It is a simple 1 per cent. solution of wood creasote in water, and like similar solutions of carbolic acid and creso'e, it is a most effective local anæsthetic, and topical dressing to burns and scalds. It is no better than the solutions of carbolic acid, or of coal-tar creasote, for this purpose, but is quite as good, so that whichever is most accessible or most convenient may be used. This creasote water, as made by the above formula—or diluted with an equal volume of water, or with more water for delicate surfaces in women and children—and applied by a means of a single thickness of thin muslin, or worn-out cotton or linen, such as handkerchief stuff, and the application renewed from time to time, as the return of pain requires it—will relieve the pain of burns and scalds in five to ten minutes, and will maintain the relief as long as the applications are properly renewed, or until the painful stage is over. It is also very effective as a local anæsthetic for general use in all painful conditions which affect the surface only, such as the pain of erysipelas. The benumbing effect of these phenols upon the skin is very promptly reached, and can be carried to almost any degree that is desirable, by simple management of the strength of the solutions and the mode of application. They are true anæsthetics to the skin, while the much lauded cocaine is not. This statement has been published so often during the past twenty years, and the treatment has been so effective in so many hands, the old and comparatively useless and hot dressings, such as carron oil, white lead ground in oil, flour, liniments, etc., or the newer application of solution of bicarbonate of sodium.—*An Ephemeris of Materia Medica, etc., March, 1885.*

**The Treatment of the Umbilical Cord** — Crede and Weber (Leipzig, in the Archiv. f. Gynak, Edin. Med. J.) set themselves to answer the question, How is bleeding from the divided cord to be obviated? and, How is inflammation and its results, of the fœtal portion, to be prevented? In the first place, they state that they are dissatisfied with the ordinary methods of tying by tape or linen; but both from clinical experience and as result of experiments made on cords post-partum, they recommend strongly the use of elastic ligatures, as suggested by Budin, and as used by them in Leipzig for the past eighteen months, with perfectly satisfactory results. The ligature used is two mm. thick, and is tightly wrapped round the cord, tied, and again taken half round and retied. As by this means the operator can be perfectly certain that there will be no bleeding, the point ligatured should be close to the skin on the cord, as, according to the writers, the shorter the portion left attached to the child, the less chance is there of traumatic inflammation. The after-treatment simply consists in keeping dry wadding round the stump, and carefully drying after the child has been bathed. Since the above treatment has been followed in the Leipzig Maternity, there have been no cases of umbilical disease.—*Journ. of Amer. Med. Ass'n.*

**Brief Note on the Treatment of Fracture of the Clavicle.**—Dr. Teller, in the N. Y. Medical Record, writes: Fracture of the clavicle is one of those ailments which are not so serious as troublesome, from the difficulty of keeping the fractured ends in apposition. All the various apparatus for that purpose have failed, and the consequence of failure is a protuberance on the point of fracture, persisting through life, unsightly if not inconvenient.

To avoid this result I propose the following plan of treatment: Turn the arm of the affected side behind the back, and bring it as far backward as possible in such manner that the dorsal side of the hand comes to lie on the opposite buttock. To retain this position, it is necessary only to fix a narrow strip of adhesive plaster around the wrist, and thence around the body, or the whole arm may be fixed on the body by means of a roller bandage from under the axilla down to the crista iliæ.

I have adopted this plan for the last two years, treating in that way about a dozen cases (with one exception, all children), and the result in every case was a speedy union without deformity. The only drawback is the difficulty of lying down, and in fact, in my first case, a girl of ten, the patient sat up in a chair day and night for two weeks, but I found after a while that the children are able to obtain perfect rest sleeping on the sound side.

**Carbolized Resin in In-growing Toe-nail.**—Dr. D. Genese, of Baltimore, writes: After using carbolized resin in dental practice for nearly ten years, I was induced to try it in a case of in-growing toe-nail of a very acute form, in which case it has given permanent relief, arresting the pain and inflammation after the first application. Since then, several cases have been treated successfully, the patients all saying relief was immediate. I have given some of the preparation to several medical gentlemen of this city, and should like to hear what success they have had with it.

It is made with Calvert's pure carbolic acid and pure French pine resin Great care is needed in not having any free acid. Messrs. Lilly, Rogers & Co. have offered to prepare the carbolized resin according to formula I have given them; and any one wishing to try this can obtain it at their store.

One drop is sufficient to apply at a time, and a pointed orange-wood stick is best to apply it with. Upon two or three applications all trouble disappears; as, after the first application has been on twenty-four hours, the nail can be lifted and a pad of lint placed under without pain.—*Maryland Med. Jour.*

**Chlorate of Potassium in Abortion.**—Dr. John W. Tradi, of Sedalia, Mo., reports a case of "Threatened Abortion Successfully treated by Chlorate of Potash." His patient had been married six or eight years and miscarried each year about the sixth month. He gave 2½ grains every three hours. The patient was in the worst possible condition, and was obliged to remain in bed the whole period of utero-gestation.

No less an authority than Karl Braun, in his recent works, speaks favorably of the use of potassium chlorate in miscarriage.

**Pilocarpia in Diphtheria.**—Dr. George Guttman, of Cronstadt, claims to have good results from pilocarpia in the treatment of diphtheria. In an experience of a year and a half, during which a large number of cases, both severe and mild, were treated, his success has been very encouraging. The drug induced abundant secretion from the mucous membrane of the throat and fauces, which brought away the false membrane. No injurious inflammation was excited, nor was there any marked tendency to depression. When symptoms of depression were observed, he alternated the medicine with stimulants in some form. The following are his formulæ:

*For Children:*

℞. Pilocarpiæ mur. . . . . . . . . . . . . . . . . . . . . . . . . gr. $\frac{1}{3}$–$\frac{2}{3}$

Pepsinæ. . . . . . . . . . . . . . . . . . . . . . . . . . . . . . . gr. i–j$\frac{1}{4}$

Acid. hydrochlor . . . . . . . . . . . . . . . . . . . . . . . . gtt. ij

Aq. dest. . . . . . . . . . . . . . . . . . . . . . . . . . . . . . ℥ ijss

M. Sig. A teaspoonful hourly.

*For Adults:*

℞. Pilocarpiæ mur . . . . . . . . . . . . . . . . . . . . . . . . gr. ss–j
Pepsinæ. . . . . . . . . . . . . . . . . . . . . . . . . . . . . . . gr. xxx
Acid hydrochlor . . . . . . . . . . . . . . . . . . . . . . . . . gtt. iij
Aquæ dest. . . . . . . . . . . . . . . . . . . . . . . . . . . . . ℥ viij
Sig. Teaspoonful hourly.—*Louisville Med. News.*

**Glycerine for Dryness of Tongue and Thirst in Febrile States.**—From a foreign exchange we learn that Surgeon Major S. K. Cotter, in a recent number of the *Indian Medical Gazette*, relates the case of a patient suffering from enteric fever who was awakened every ten minutes by the dryness of his tongue, which was parched and covered with sordes. The tongue was painted with glycerine frequently, and the result was that at the first trial the patient slept almost comfortably, waking up about every two hours with the tongue feeling dry, but not really dry to the touch; after renewed application of the glycerine he at once slept again. In six other cases it has been tried and found satisfactory. Surgeon Major Cotter does not attempt to decide whether it acts by increasing secretion from the mucous membrane, dissolving the sordes, or making an artifical coating, but, in whatever way it acts, its benefits is vouched for when the tongue is parched during any disease.—*Medical and Surgical Reporter.*

**The Influence of Diet on Headache.**—Haig (Practitioner) reports at length a peculiar case of migraine which had long resisted treatment, and was finally cured only by strict adherence to a vegetable diet. Meat seemed to act upon the patient as a veritable poison.—*N. Y. Medical Journal.*

**Antidote for Iodoform.**—Dr. Behring found tablespoonful hourly doses of a twenty-per-cent solution of bicarbonate of potassium to act as a prompt antidote in iodoform poisoning.—*Louisville Medical News.*

**An Antipyretic Formula.**—The following is recommended by Dr. de Giovanni as a substitute for quinine, in which the latter is ineffective or injurious in its effects (*Rivista Clinica*, No. 8, 1884): Ergotine (Bonjean), fifteen grains; tincture of valerian, half an ounce; water, three ounces; to be taken during the height of the fever or in the period of remittance. Usually the good effects are obtained after one or two doses, but the remedy should be continued for some time after the subsidence of the fever. In rebellious cases, the author substituted with benefit cherry-laurel water, one-half to one drachm, for the valerian The best results were obtained in intermittent fevers, but good effects were seen also in remittents, in the hectic of incipient phthisis, and even in puerperal fever.—*N. Y. Med. Record.*

**Oranges for a Deficient Milk Secretion.**—A writer in *N. C. Medical Journal* says: "In December a relative living in Florida sent Mrs. R. a box of oranges. Two days after their reception she noticed an increase in the flow of milk, and expressed the opinion that eating oranges caused it. I had never heard of this fruit as a lactagogue, and asked her to suspend the eating of oranges for a few days to test the matter. This she did, and found that the milk did not flow so freely. She commenced the use of oranges regularly, three a day, as long as her box lasted, with a plentiful flow of milk all the while.

Mrs. R. is now visiting in Florida, and writes me that if the orange crop holds out, she has no fears as to the baby's support.

**The Disadvantages of Glycerin as a Solvent.**—Vigier (L'Union pharm.; "Memorabil.") calls attention to the fact that, although glycerin is a valuable solvent, it should not be allowed to supersede fats in the preparation of ointments, for it is not absored through the skin. As a result of numerous experiments upon himself, he states that strong solutions of potassium iodide, morphine, atropine, and corrosive sublimate in glycerin not only fail to produce constitutional effects when rubbed into the skin, but do not appear in the urine; whereas, when incorporated with fat in like proportion, they are rapidly absorbed.—*New York Medical Journal.*

**Pepsin as a Solvent of Blood-Clots in the Bladder.**—The editor of the *Northwestern Lancet* calls attention to a novel use of pepsin in surgery. In a case of distended bladder, when clotted blood prevented evacuation, he dissolved a scruple of Jensen's crystal pepsin in an ounce of warm water, and in less than twenty minutes had the satisfaction of seeing the patient pass a full stream of urine and disintegrated blood.—*Med. Times.*

**Sulphide of Calcium** is said to have an almost specific action on diseases of the ear, accompanied by suppuration, as otitis, purulent catarrh, etc. It should be given in small and repeated doses, in the solid form, being too unstable when dissolved.—*Courier-Record.*

